# Momentum-resolved observations of the phonon instability driving geometric improper ferroelectricity in yttrium manganite

**DOI:** 10.1038/s41467-017-02309-2

**Published:** 2018-01-02

**Authors:** Dipanshu Bansal, Jennifer L. Niedziela, Ryan Sinclair, V. Ovidiu Garlea, Douglas L. Abernathy, Songxue Chi, Yang Ren, Haidong Zhou, Olivier Delaire

**Affiliations:** 1 Department of Mechanical Engineering and Materials Science and Department of Physics, Duke University, Durham, NC 27708 USA; 2 Materials Science and Technology Division, Oak Ridge National Laboratory, Oak Ridge, TN 37831 USA; 3 Department of Physics and Astronomy, University of Tennessee, Knoxville, TN 37996 USA; 4 Neutron Scattering Division, Oak Ridge National Laboratory, Oak Ridge, TN 37831 USA; 5 Advanced Photon Source, Argonne National Laboratory, Argonne, IL 60439 USA

## Abstract

Magnetoelectrics offer tantalizing opportunities for devices coupling ferroelectricity and magnetism but remain difficult to realize. Breakthrough strategies could circumvent the mutually exclusive origins of magnetism and ferroelectricity by exploiting the interaction of multiple phonon modes in geometric improper and hybrid improper ferroelectrics. Yet, the proposed instability of a zone-boundary phonon mode, driving the emergence of ferroelectricity via coupling to a polar mode, remains to be directly observed. Here, we provide previously missing evidence for this scenario in the archetypal improper ferroelectric, yttrium manganite, through comprehensive scattering measurements of the atomic structure and phonons, supported with first-principles simulations. Our experiments and theoretical modeling resolve the origin of the unusual temperature dependence of the polarization and rule out a reported double-step ferroelectric transition. These results emphasize the critical role of phonon anharmonicity in rationalizing lattice instabilities in improper ferroelectrics and show that including these effects in simulations could facilitate the design of magnetoelectrics.

## Abstract (web summary)

The improper ferroelectric mechanism of YMnO_3_ has been difficult to observe directly but is important for understanding its other interesting properties such as multiferroicity. Bansal et al. use neutron scattering and ab initio calculations to show that phonons drive the transition, confirming earlier theories.

## Introduction

The prospect of controlling magnetic order via electric fields, and vice-versa, is captivating broad interest in developing multiferroic materials, for applications ranging from next-generation data storage to sensing and energy conversion^1–12^. Because magnetism and displacive ferroelectricity do not normally coexist (as they are normally associated with partially filled vs. empty transition metal orbitals^13^, respectively), a quest for mechanisms enabling compatibility has flourished^9–12,14^. A promising avenue was identified in so-called geometric improper and hybrid improper ferroelectricity^7,15–20^ co-existing with magnetism^7,8,12,15,17,19,21,22^. It is well established that in “proper” displacive ferroelectric (FE) transitions, the polarization (*P*) emerges from the condensation of a soft polar mode at the zone-center (wavevector *q* = 0), e.g., in BaTiO_3_, KNbO_3_, BiFeO_3_^14,18,23^. However, the mechanism underlying “improper” FE transitions, exemplified by the multiferroic hexagonal manganite YMnO_3_, still remains unconfirmed^15,18,24^. It is proposed that such transitions arise from anharmonic interactions between an unstable non-polar phonon mode at finite wavevector (*q* ≠ 0) and a soft but stable zone-center polar mode^15^. The improper FE transition is key to realizing topological (anti)vortices^12,25^, and offers an opportunity to tune *q* ≠ 0 non-polar distortions via electric fields to engineer multifunctional properties not accessible in proper FEs^7,16–18,20^.

Despite extensive efforts, using a wide range of experimental techniques^26–35^ and theoretical simulations^15,26,27,33,35,36^, no reported experiments or simulations, to our knowledge, have previously probed the *q*- and *T*-dependence across $T_{{\mathrm{FE}}} \simeq 1260$ K of the proposed *q* ≠ 0 phonon instability thought to drive the FE transition in YMnO_3_. This is largely because of the experimental difficulty in observing the relevant phonon modes, and limitations of the harmonic approximation in first-principles simulations. Experimental studies with Raman and infrared (IR) spectroscopy^26,29,34^ were intrinsically limited to *q* = 0. Surprisingly, recent measurements of the momentum-integrated phonon density of states (DOS) using inelastic neutron scattering (INS) on polycrystalline YMnO_3_ showed little change across *T*_FE_^35^. However, as we show here, the behavior of key phonon modes is obscured in the DOS, which averages over all wavevectors. The *q*-resolved INS measurements of Petit et al.^37^, on the other hand, focused only on the low-*T* antiferromagnetic transition (~75 K), leaving open questions about the mechanism of the phonon instability across *T*_FE_.

Moreover, the structural evolution across the FE transition remains uncertain. It is known that YMnO_3_ crystallizes in the paraelectric (PE) *P*6_3_/mmc (#194) at high temperature, and transforms to the FE *P*6_3_cm space group (#185) on cooling below *T*_FE_^31^. But conflicting reports of a double-step^27,28,31,34,38^ vs. single-step transition^15,32,33^ have impeded a coherent understanding of the transition sequence. Previous X-ray and neutron powder diffraction studies have remained inconclusive^28,31,39^, calling for single-crystal measurements. Besides, diffraction experiments alone, lacking energy resolution, cannot resolve atomic dynamics critical in improper ferroelectrics.

Here, we report decisive measurements of momentum-resolved phonon dispersions in YMnO_3_ with INS, including the behavior across the FE transition, as well as single-crystal neutron and X-ray diffraction (XRD) measurements of the structural distortion. INS is uniquely powerful to map phonon dispersions across the entire Brillouin zone, providing a stringent test of microscopic theoretical models. In addition, we performed first-principles phonon simulations with density functional theory (DFT), in both the FE and PE phases, explicitly including the anharmonic renormalization of phonon dispersions at high temperature. The excellent agreement between our experiments and simulations enables us to elucidate the mechanism of the improper FE transition, and determine the precursor instability in the PE phase. Our results establish the single-step nature of the transition and directly reveal the unstable mode at the K_3_ zone-boundary point in the PE phase, which condenses at *T*_FE_. We model the coupling between primary- and secondary-order parameters to rationalize the temperature dependence of *P*, which is strikingly different from that in proper ferroelectrics [*P* ∝ (*T*_c_ − *T*)^0.5^]^14^.

## Results

### Structural phase transition and lattice distortions

**Fig. 1:**
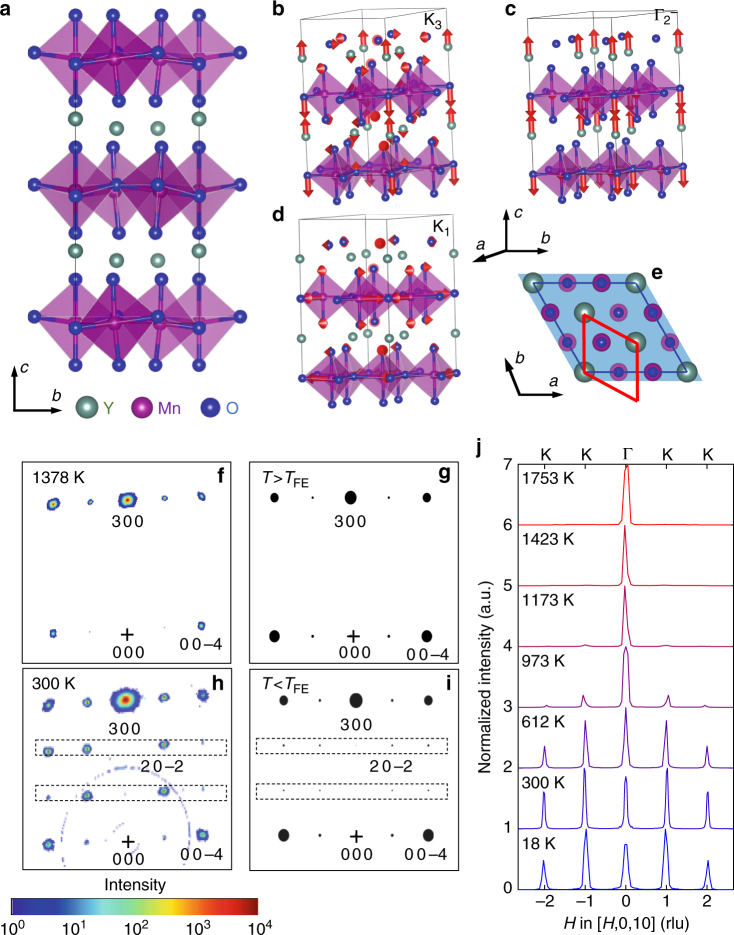
Structural phase transition and lattice distortions across *T*_FE_. **a** The low-*T* FE structure (*P*6_3_cm), showing tilts and rotations of MnO_5_ polyhedra and displacement of Y atoms along *c*. Y, Mn, and O atoms are represented by light green, purple, and blue color, respectively. **b**–**d** The decomposition of the total distortion into the **b** K_3_ mode, **c** ${\mathrm{\Gamma }}_2^ -$ mode, and **d** K_1_ mode. **e** Trimerization of the unit cell with rotation across *T*_FE_: the PE and FE phase unit cells are shown with red and blue lines. **f**, **h** Single-crystal XRD maps (*E*_i_ = 105.091 keV) measured at 1378 K (**f**), and 300 K (**h**) in (*H*0*L*) reciprocal plane. **g**, **i** are simulated diffraction patterns for the PE and FE phases. Above *T*_FE_ ≃ 1260 K, the *H*0*L* reflections disappear for *H* ≠ 3*n*, e.g., (2 0 −2). **j** Normalized intensity (maximum value normalized to 1) of Bragg peaks (0, 0, 10), (±1, 0, 10), and (±2, 0, 10) as function of temperature, obtained by integrating INS data over −1 ≤ *E* ≤ 1 meV. The (1, 0, 10) and (2, 0, 10) Bragg peaks are seen at 1173 K and below due to the trimerization of the unit cell. The high symmetry points (Γ and K) denoting the path above (**j**) refer to the high-*T* PE phase setting. The curves are offset for clarity. Panels **a**–**e** are plotted with VESTA^51^

Our XRD and neutron diffraction measurements on single crystals reveal a clear, single-step phase transition with trimerization of the unit cell at *T*_FE_ ~ 1260 K. Figure 1a–d illustrates the structure modulation settling across *T*_FE_, with buckling of the yttrium plane along *c* and tilts of MnO_5_ polyhedra. Note the tripling of unit cell volume (trimerization) from PE to FE (Fig. 1e), leading to superlattice Bragg peaks in the FE phase (compare panels Fig. 1g, i). The transition temperature is confirmed by our calorimetry measurements (Supplementary Fig. 1) and is in good agreement with previous reports^31–33^. Group theoretical analysis^40^ showed that the FE transition is enabled by four symmetry-adapted modes of the PE phase: ${\mathrm{\Gamma }}_1^ +$ (identical representation), ${\mathrm{\Gamma }}_2^ -$ (zone-center polar mode), K_1_, and K_3_ (phonon modes at *q* = 1/3, 1/3, 0 in the PE phase). The latter three are illustrated in Fig. 1b–d. Figure 1f, h, j show single-crystal XRD and neutron diffraction patterns, respectively, measured in the (*H*0*L*) reciprocal plane across *T*_FE_. All reciprocal space indexing is done with reference to the FE unit cell. As one can observe from Fig. 1f–j, *H*0*L*-type superlattice reflections for *H* ≠ 3*n* (*n* integer) appear on cooling below 1300 K, revealing both K_3_ and K_1_ lattice distortions. Fennie and Rabe^15^ pointed out that K_3_ is the more potent distortion and should be the primary order parameter, inducing trimerization, which we confirm with INS (as shown below). Since the amplitude of the K_1_ mode (0.03 Å) is much smaller than that of K_3_ (0.93 Å)^15,40^, the superlattice peak intensities are primarily determined by the K_3_ modulation. The stable zone-center polar mode, ${\mathrm{\Gamma }}_2^ -$, lowers the lattice symmetry from *P*6_3_/mmc to *P*6_3_mc (Space group: 186) but does not lead to new Bragg peaks below *T*_FE_.

### Dynamics of the non**-**polar zone-boundary K_3_ distortion

**Fig. 2:**
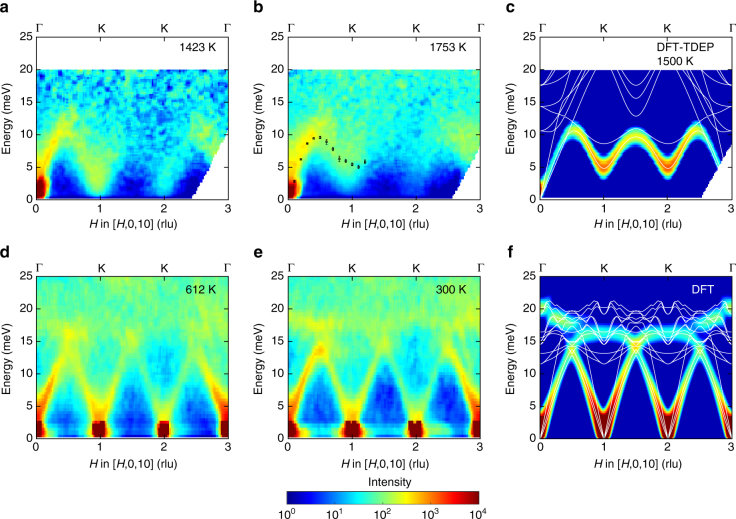
Dynamics and condensation of the K_3_ distortion across *T*_FE_. Phonon dispersions along [*H*, 0,10] direction measured at *T* = **a** 1423, **b** 1753, **d** 612, and **e** 300 K are compared with intensity calculated from DFT model, *χ*″(**Q**, *E*), for **c** 1500 K and **f** 0 K DFT simulations (white lines in **c**, **f** are the calculated phonon dispersions along the [*H*, 0, 10]). The (1, 0, 10) and (2, 0, 10) Bragg peaks at 612 and 300 K are due to the trimerization of the unit cell. In **e**, the phonon dispersion obtained by fitting the scattering intensity is shown as black markers with error bars (one standard deviation on either side of the peak value). The INS signal was integrated over a range −0.2 ≤ **Q**_⊥_ ≤ 0.2 rlu for directions perpendicular to [*H*, 0, 10]. The high symmetry points (Γ and K) denoting the path above panels refer to the PE phase setting (K becomes Γ in the FE phase). The small difference between the measurements and simulations of phonon intensity near (2, 0, 10) is due to slightly different theoretical atomic coordinates (and consequently structure factor), and possible effects of sample rotation in the beam. The data in **a**, **b** and **d**, **e** were measured on HYSPEC (phonon annihilation) and ARCS (phonon creation) spectrometers, respectively

Using INS data, we now investigate the dynamics of the primary K_3_ distortion across *T*_FE_ by following the out-of-plane (*c*) polarized transverse acoustic (TA) branch along the [*H*, 0, 10] reciprocal lattice direction, whose polarization overlaps with K_3_. Figure 2a, b, d, e shows maps of the dynamical susceptibility from INS, $\chi {\prime\prime}({\bf{Q}},E) = S({\bf{Q}},E){\mathrm{/}}\left( {n_s + \frac{1}{2} \pm \frac{1}{2}} \right)$ (Methods), at 300, 612, 1423, and 1753 K (see Supplementary Fig. 2 for additional temperatures). The INS data are compared with our 0 K and finite temperature DFT simulations in Fig. 2c, f. Here, **Q** denotes the wave-vector transfer, *E* the energy transfer, and *k*_B_ is the Boltzmann’s constant. We write **Q** = ***τ***_*HKL*_ + **q**, with ***τ***_*HKL*_ the nearest reciprocal lattice vector, and **q** the reduced phonon wave vector. Note that Γ − K (***τ*** + [*HH*0]) in the PE phase is equivalent to Γ − M (***τ*** + [*H*00]) in the FE phase. Above *T*_FE_, the TA branch shows pronounced dip at K points $\left( {H \in {\Bbb Z},H \ne 3n} \right)$ but retains a finite gap (Fig. 2a, b). At the highest *T* measured in the PE phase, the TA branch rises from *H* = 0 up to ~10 meV at *H* = 0.5, then curves back down to *E* ~ 5 meV at (1, 0, 10). Phonon energies obtained by fitting Gaussians peaks to constant-*q* cuts are shown with black markers in Fig. 2b. While a clear gap is seen at K points for *T* > *T*_FE_, the excitation is also considerably broadened in energy, which indicates that the K_3_ oscillations are strongly damped in the PE phase, reflecting a large anharmonicity. The FE transition corresponds to condensation of the zone-boundary K_3_ modes at *T*_FE_ closing the gap and leading to the formation of the superlattice Bragg peaks at (1, 0, 10) and (2, 0, 10) as shown in Figs. 1j and 2d, e. The absence of superlattice Bragg peaks above *T*_FE_ indicates the absence of static, long-range correlations. However, precursor dynamical correlations exist above *T*_FE_ at K points, and these correlations are clearly seen in constant-*E* maps at low energy, shown in Supplementary Fig. 3. We note that it is important to separate low-energy dynamical correlations from static correlations, as was done here, and that this is harder to do in conventional diffraction measurements, which integrate over both signals.

The striking experimental behavior of the K_3_ soft mode is quantitatively captured in our DFT lattice dynamics simulations. As shown in Supplementary Fig. 4, phonon dispersions calculated using the harmonic approximation result in an imaginary (unstable) branch for the K_3_ distortion in the PE phase (represented as a negative frequency), which is most pronounced at K points. This instability is renormalized by anharmonicity in our ab initio molecular dynamics (AIMD) simulations^41^, leading to a stabilized branch at 1500 K, as shown in Fig. 2c and Supplementary Fig. 5a. These are, to our knowledge, the first physically realistic first-principles simulations of the lattice dynamics of the PE phase in improper ferroelectrics, capturing renormalization of the unstable phonons by anharmonic effects at high *T*. The anharmonic DFT simulations correctly predict the finite energy gap opening (~4.8 meV) at [1, 0, 10] and [2, 0, 10] in the PE phase and are in quantitative agreement with our experimental values of 3.41 ± 0.76 and 5.44 ± 0.22 meV at 1423 and 1753 K, respectively. However the computed phonon DOS has only a small change across *T*_FE_ explaining the apparent lack of effect reported in previous measurements^35^ (Supplementary Fig. 5b, d). Although stabilized at 1500 K, the computed TA branch shows a pronounced dip at K points similar to INS measurements, reflecting the precursor instability at *T* > *T*_FE_. This behavior is understood as a renormalization of the effective K_3_ potential by the phonon bath at high *T*, as illustrated in Supplementary Fig. 6a. We emphasize that Raman and IR spectroscopy cannot access the continuous condensation of the zone boundary *q* ≠ 0 instability in the parent PE phase, while INS provides the full dispersions across the Brillouin zone, in both phases. Even in the FE phase, at the superlattice Γ points, only one of the two optical modes (*A*_1_ and *B*_2_) overlapping with the parent K_3_ distortion is accessible^26,29,34^, since the *B*_2_ mode is silent in both Raman and IR. On the other hand, both optical phonon branches are accessible with INS (*A*_1_ is near ~20 meV at (0, 0, 10), *B*_2_ is near ~15 meV at (1, 0, 10), see Fig. 2e, f and Supplementary Fig. 2a).

We now follow the dynamics of the K_3_-derived modes on cooling in the FE phase. Figure 2d, e directly reveal low-*q* acoustic modes emanating from the new superlattice peaks whose intensity increases with the growing lattice distortion on cooling. The *T*-dependence of K_3_-like phonons can be determined from the constant-*Q* cuts along [*H*, 0, 10]. For **q** away from the new Bragg peaks (e.g., (0.4, 0, 10)), the TA modes in FE phase show a recovery stiffening (increase) from 9.84 ± 0.07 meV at 1173 K to 12.75 ± 0.05 at 18 K on cooling as the K_3_ distortion increases (Supplementary Fig. 2h). This stiffening on cooling is also consistent with our DFT simulations of the temperature evolution of the K_3_ potential energy, where the curvature at the minimum increases on cooling below *T*_FE_: $\left( {\partial ^2E{\mathrm{/}}\partial Q_{{\mathrm{K}}_{\mathrm{3}}}^2} \right)^{1{\mathrm{/}}2} = K_{{\mathrm{K}}_{\mathrm{3}}}^{{\mathrm{eff}}^{1/2}} \propto \omega _{{\mathrm{K}}_{\mathrm{3}}}^{{\mathrm{eff}}}$, with $K_{{\mathrm{K}}_3}^{{\mathrm{eff}}}$ and $\omega _{{\mathrm{K}}_3}^{{\mathrm{eff}}}$ the effective stiffness and phonon energy of K_3_ lattice distortion, as shown in Supplementary Fig. 6a. We emphasize that by mapping the 4-D *S*(**Q**, *E*), we were able to establish the absence of additional instabilities at any other *q* points. This is a critical point to benchmark theoretical predictions in this strongly anharmonic system.

### Dynamics of the K_1_ distortion

**Fig. 3:**
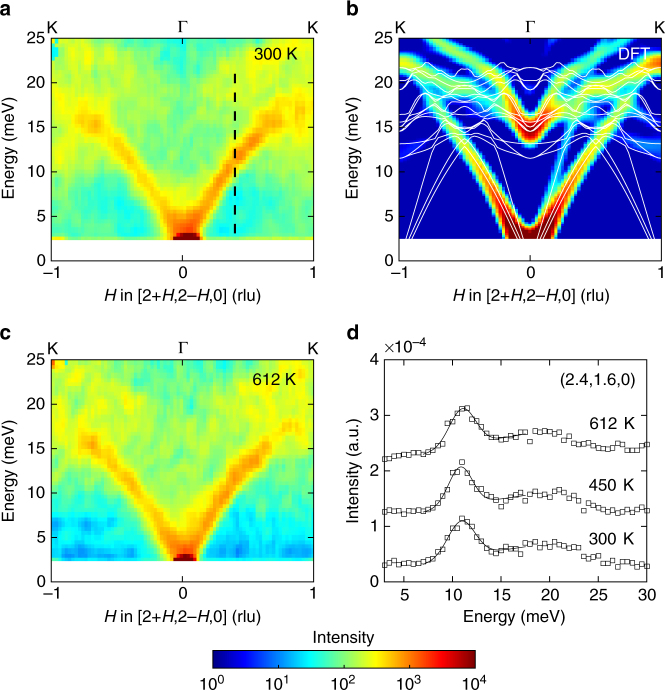
Dynamics of the K_1_ distortion in the FE phase. **a**, **c** *χ*″(**Q**, *E*) along the [2 + *H*, 2 − *H*, 0] direction at *T* = 300, and 612 K compared with calculated intensity from DFT simulations (white lines are calculated phonon dispersion curves in **b**). **d** 1D spectra at *Q* = (2.4, 1.6, 0) (black dotted line in **a**). The integration range in **a**–**c** along perpendicular *Q* directions is from −0.2 to 0.2 rlu, while for 1D cuts, the integration range along *H* is from 0.35 to 0.45 rlu. To improve the statistics of 1D cuts, data at equivalent point *Q* = (1.6, 2.4, 0) are summed with data at *Q* = (2.4, 1.6, 0). Γ and K high symmetry points in **a**–**c** refer to the PE phase notation. Error bars are smaller than the size of markers. The data are measured on ARCS instrument. The offset for different temperature points is for clarity

Additional INS measurements were performed in the (*HK*0) scattering plane in order to investigate the dynamics of K_1_-like phonons, whose eigenvectors are parallel to the basal plane (Fig. 1d). The dynamics of the K_1_ distortion were measured along [H$\overline {\mathrm{H}}$0] around ***τ*** = (220). Figure 3a–c shows *χ*″(**Q**, *E*) along the [2 + *H*, 2 − *H*, 0] at 300 and 612 K, compared with our DFT simulations. The simulations and measurements are in good agreement, and both show that the K_1_-like TA branches emanating from (1, 3, 0) and (3, 1, 0) are exceedingly weak. While faint superlattice Bragg peaks are indeed observed at (1, 3, 0), (3, 1, 0), and (4, 0, 0) in the FE phase (Supplementary Fig. 7), these are much weaker than K_3_-modulation peaks at (1, 0, 10) and (2, 0, 10), and the intensity of TA phonons emanating from them was not observable. The *T*-dependence of a nearby TA mode at (2.4, 1.6, 0), of similar character as the K_1_ lattice distortion, is shown in Fig. 3d. We find no change in the energy of this mode between 300 (10.77 ± 0.07 meV) and 612 K (10.89 ± 0.06 meV), within the instrumental energy resolution. For comparison, in the same temperature range, a mode probing the K_3_ distortion in the FE phase shows a pronounced change of 2.07 ± 0.17 meV. This result is also explained by our first-principles simulations, which yield very similar phonon energies for both the PE and FE phases for $\left| {{\bf{Q}} - {\boldsymbol{\tau }}_{220}} \right| < 0.5$ along [2 + *H*, 2 − *H*, 0], and compatible with group theoretical analyses that showed the amplitude of the K_1_ distortion, $Q_{{\mathrm{K}}_1}$, to be significantly smaller than that of $Q_{{\mathrm{K}}_3}$ and $Q_{{\mathrm{\Gamma }}_2^ - }$ ^15,40^.

### Dynamics of the zone-center polar ${\mathrm{\Gamma }}_2^ -$ distortion

**Fig. 4:**
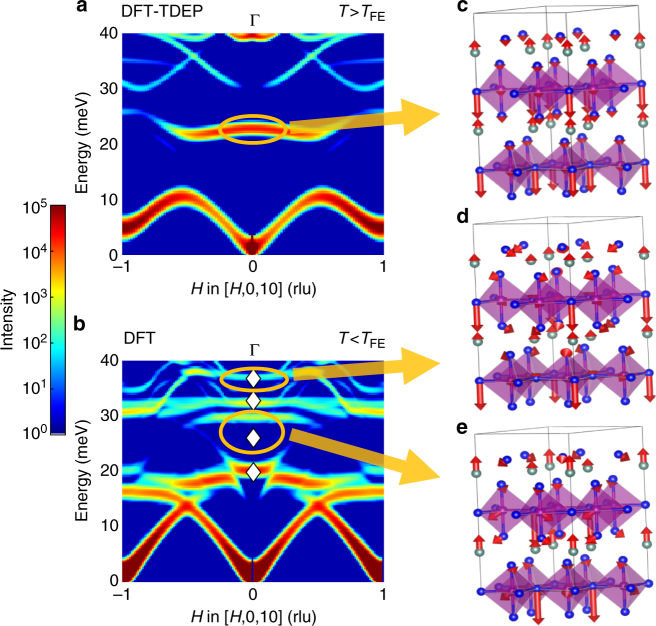
Dynamics of the polar ${\mathrm{\Gamma }}_2^ -$ distortion in the PE and FE phases. **a**, **b** Intensity, *χ*″**(Q**, *E*), of phonon modes along [*H*, 0, 10] direction calculated from 0 and 1500 K DFT simulations. Phonon eigenvectors of the ${\mathrm{\Gamma }}_2^ -$ distortion in the **c** PE, and **d**, **e** FE phase. The ${\mathrm{\Gamma }}_2^ -$ distortion in the PE phase at 22.85 meV overlaps with two modes in the FE phase and is present at 29.72 and 37.01 meV. The white diamonds are Raman measurements^29,34^. **c**–**e** are plotted with VESTA^51^

Further, our first-principles lattice dynamics simulations, validated against INS measurements, are critical in clarifying the behavior of the ${\mathrm{\Gamma }}_2^ -$ phonons. In the FE transition, the unstable K_3_ distortion creates a geometric field that couples to the polar ${\mathrm{\Gamma }}_2^ -$ distortion to induce the finite *P*, as proposed in ref. ^15^ and also shown in Supplementary Fig. 6. However, previous simulations have not resolved the evolution of the ${\mathrm{\Gamma }}_2^ -$ mode across the FE transition. Figure 4 shows *χ*″(**Q**, *E*) along [*H*, 0, 10] for *H* near zero, where the contribution from the ${\mathrm{\Gamma }}_2^ -$ distortion is significant. The ${\mathrm{\Gamma }}_2^ -$ modes in both phases are indicated with ellipses, and their phonon eigenvectors are illustrated in Fig. 4c–e. We note that, because the ${\mathrm{\Gamma }}_2^ -$ intensity in the low-*T* phase is three orders of magnitude weaker than the acoustic modes, its signal is difficult to discriminate from the background in our INS measurements. The calculated ${\mathrm{\Gamma }}_2^ -$ mode frequency in the PE phase is at *E* = 22.85 meV, and overlaps with two phonon modes in the FE phase (also predicted by Prikockyte et al.^36^, but limited to the FE phase at 0 K). The energies of the resulting modes are 29.72 and 37.01 meV according to our 0 K DFT simulations, in good agreement with Raman spectroscopy: 26.04 meV at 15 K^29^ and 36.82 meV at room temperature^34^, respectively.

### Improper FE coupling of polar and non-polar distortions

**Fig. 5:**
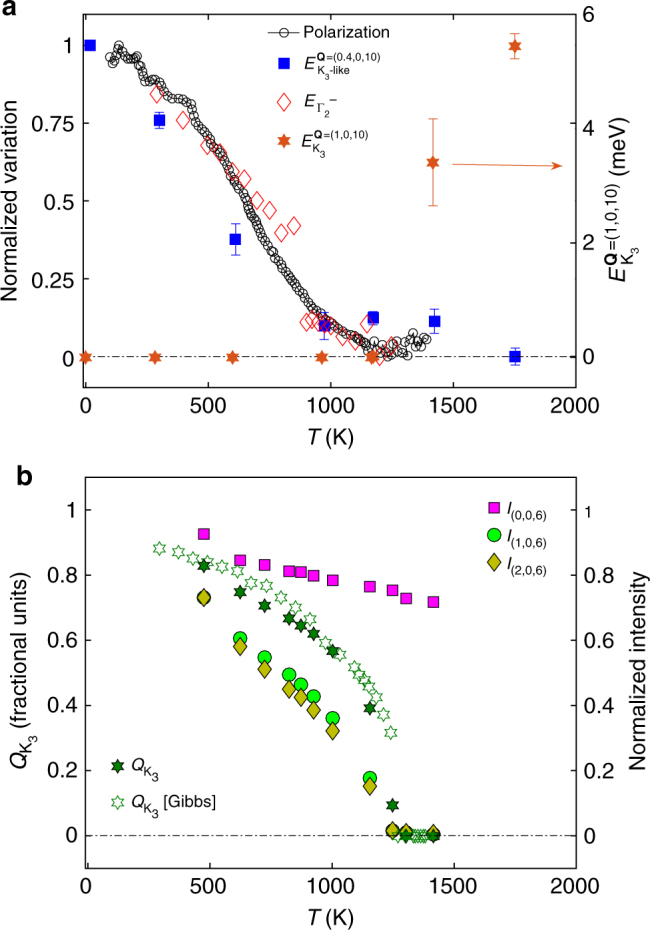
Lattice distortion, dynamics, and polarization. **a** Normalized variation of polarization, and phonon energy of (0.4, 0, 10) and ${\mathrm{\Gamma }}_2^ -$ mode of YMnO_3_ as a function of temperature. On the right side *y*-axis, we show the phonon energy of the K_3_ mode as a function of temperature extracted by fitting the constant-**Q** scan at (1, 0, 10)—K point of the PE phase. K_3_ mode condenses at *T*_FE_, and becomes a Bragg peak in the FE phase. The phonon energy at **Q** = (0.4, 0, 10) corresponds to stiffening of the TA mode from continuation of the K_3_-like distortion in the FE phase. The *T*-dependence of *P* and $E_{{\mathrm{\Gamma }}_2^ - }$ is taken from Fig. 1b in ref. ^33^, and Fig. 4b (labeled as *A*_1_(*TO*_4_)) in ref. ^34^, respectively, while the *T*-dependence of $E_{{\mathrm{K}}_{\mathrm{3}} - {\mathrm{like}}}^{{\mathbf{Q}} = (0.4,0,10)}$ is extracted from fitting the TA phonon peak as shown in Supplementary Fig. 2h. The data have been normalized between 0 and 1 using $x_{\mathrm{nor}} = \frac{{x - {\mathrm{min}}(x)}}{{{\mathrm{max}}(x) - {\mathrm{min}}(x)}}$. All error bars are one standard deviation on either side of the peak value. **b** Normalized integrated Bragg intensity of (0, 0, 6), (1, 0, 6), and (2, 0, 6) peaks as observed at multiple temperatures using HB-3 triple-axis spectrometer, and amplitude of the K_3_ distortion, $Q_{{\mathrm{K}}_3}$, compared with experimental measurements of Gibbs et al.^31^ The intensity of Bragg peaks (1, 0, 6) and (2, 0, 6) is normalized to 0.73 (factor obtained from polarization value at *T* = 473 K in Fig. 5a). Error bars are smaller than the size of markers

Based on measurements and simulations, we can now rationalize the experimental polarization data from Lilienblum et al.^33^ and clarify the controversy surrounding the single- vs. double-step character of the FE transition. In Fig. 5a, we plot the temperature dependence of *P*, and energy of ${\mathrm{\Gamma }}_2^ -$ Raman phonons (from Bouyanfif et al.^34^) and K_3_-like distortion from our INS data. As one can observe, although the distortion amplitude $Q_{{\mathrm{K}}_3}$ increases quickly below *T*_FE_ (Fig. 5b), the phonon energy of K_3_-derived modes, $E_{{\mathrm{K}}_{\mathrm{3}} - {\mathrm{like}}}$, shows a much more gradual rise, following the temperature dependence of *P* (Fig. 5a). The phonon energy of ${\mathrm{\Gamma }}_2^ -$ distortion, $E_{{\mathrm{\Gamma }}_2^ - }$, also follows a trend similar to that of *P*, which is expected, given that $E_{{\mathrm{\Gamma }}_2^ - }$ is roughly linear in $Q_{{\mathrm{\Gamma }}_2^ - }$ (Supplementary Fig. 8c) and $Q_{{\mathrm{\Gamma }}_2^ - }$ is directly proportional to *P*. This unusual temperature dependence of $Q_{{\mathrm{\Gamma }}_2^ - }$, $E_{{\mathrm{\Gamma }}_2^ - }$, $E_{{\mathrm{K}}_{\mathrm{3}} - {\mathrm{like}}}$, and *P* implies *T*-dependent coupling constants between $Q_{{\mathrm{K}}_3}$ and $Q_{{\mathrm{\Gamma }}_2^ - }$, i.e., *η*(*T*) and *ζ*(*T*), with coupling increasing from small magnitude to DFT values calculated by freezing K_3_ and ${\mathrm{\Gamma }}_2^ -$ distortions at 0 K ($E \propto \zeta (T)Q_{{\mathrm{K}}_3}^2Q_{{\mathrm{\Gamma }}_2^ - }^2 + \eta (T)Q_{{\mathrm{K}}_3}^3Q_{{\mathrm{\Gamma }}_2^ - } + \gamma Q_{{\mathrm{\Gamma }}_2^ - }^2 + \ldots$, see Supplementary Fig. 8b, d and Methods section for more details). On the other hand, as shown by Fennie and Rabe^15^, *T*-independent coupling constants would lead to $Q_{{\mathrm{\Gamma }}_2^ - } \propto Q_{{\mathrm{K}}_3}\forall T < T_{{\mathrm{FE}}}$ (since $Q_{{\mathrm{K}}_3}$ increases suddenly below *T*_FE_, and $Q_{{\mathrm{\Gamma }}_2^ - } \propto Q_{{\mathrm{K}}_3}$ for large $Q_{{\mathrm{K}}_3}$) and thus cannot account for the experimental trend of $E_{{\mathrm{\Gamma }}_2^ - }$ or *P*. The weak coupling between $Q_{{\mathrm{K}}_3}$ and $Q_{{\mathrm{\Gamma }}_2^ - }$ at *T*_FE_ also confirms that the FE phase transition is indeed driven by the K_3_ instability as the primary order parameter, while $Q_{{\mathrm{\Gamma }}_2^ - }$ is a secondary order parameter. We note that our analysis of $Q_{{\mathrm{\Gamma }}_2^ - }(T)$ is markedly different from the double-step model of Gibbs et al.^31^ based on powder diffraction, whose result for *P*(*T*) was contradicted by the measurements of Lilienblum et al.^33^ Rather, our single-crystal results indicate that the apparent second phase transition between 900 and 1100 K^28,31,34,38^ is likely due to the gradual increase in coupling between $Q_{{\mathrm{K}}_3}$ and $Q_{{\mathrm{\Gamma }}_2^ - }$, and associated changes in phonon dynamics. The nature of *T*-dependent coupling constants is entirely consistent with the topological nature of improper ferroelectricity, where topological defect vortices with continuously varying phase adopt a discrete *Z*_6_ symmetry at a critical value of coupling strength^33,42^, and enable a coupled response of the K_3_ and ${\mathrm{\Gamma }}_2^ -$ modes^15^.

In summary, our comprehensive INS and XRD measurements on single crystals, combined with first-principles lattice dynamics simulations, including anharmonic effects, provide previously missing direct evidence of the mechanism of the geometric improper FE transition in YMnO_3_. Our data and analysis resolve an on-going controversy concerning the single- vs. double-step nature of the FE transition, and account for the unusual *T*-dependence of polarization. Our detailed study of atomic structure and dynamics across the FE transition not only validates but also refines the proposed transition path and quantitatively accounts for the origin of the polarization. Anharmonic DFT simulations of phonons at finite *T*, validated against INS measurements, provides a pathway to understand the unstable zone-boundary phonons central to geometric ferroelectrics in both “improper” and “hybrid improper” FE classes. These results and insights are general and applicable to a broad range of FE transitions resulting from anharmonic phonon–phonon interactions, and open a route toward the rational design of ferroelectrics and magnetoelectrics with desirable characteristics.

## Methods

### Sample growth

Single crystals of YMnO_3_ were grown by the floating-zone technique. The feed and seed rods for the crystal growth were prepared by solid-state reaction. Stoichiometric mixtures of Y_2_O_3_ and Mn_2_O_3_ were ground together and calcined in air at 1100 °C for 24 h. It was then reground again into a powder and pressed into a 6-mm-diameter 60-mm rod under 400 atm hydrostatic pressure, which were finally sintered at 1200 °C for 20 h. The crystal growth was carried out in air in an IR-heated image furnace with a growth rate of 4 mm h^−1^. Small pieces of the single crystals were ground into fine powder for XRD, which shows the pure phase of hexagonal YMnO_3_.

### Measurement procedures

Single-crystal XRD and INS experiments were carried out to probe the structure and lattice dynamics across *T*_FE_. XRD was performed on a 5 × 5 × 1 mm crystal with hard X-rays (*E*_i_ = 105.091 keV) at beamline 11-ID-C at the Advanced Photon Source. The crystal was mounted in a Linkam TS1500 stage, with the sample in air (Supplementary Fig. 9d). Single-crystal INS experiments were performed using the time-of-flight (TOF)-wide angular-range chopper spectrometer (ARCS) and the hybrid spectrometer (HYSPEC) at the spallation neutron source (SNS), and triple-axis spectrometer HB-3 at the high-flux isotope reactor (HFIR) at Oak Ridge National Laboratory^43^. For TOF measurements on ARCS, we used a closed-cycle helium refrigerator and a low-background resistive furnace for 18 ≤ *T* ≤ 300 K, and 300 < *T* ≤ 612 K, respectively, with an oscillating radial collimator. Two samples, each of mass ~3 gm, were co-aligned on an Al mount. The mosaic of co-aligned samples was <1.5° in *H*0*L* and 2° in *HK*0 scattering plane (Supplementary Fig. 9b, c). For high-*T* (612 < *T* ≤ 1753 K) TOF INS measurements at HYSPEC, and all triple-axis measurements, we used a high-temperature furnace with an air atmosphere to minimize oxygen vacancies at elevated temperature. A single-crystal piece of mass ~3 gm was mounted in *H*0*L* scattering plane on an Al_2_O_3_ post with Pt wires both of which are stable for our probing temperature range (Supplementary Fig. 9a). The sample mosaic was <0.75°. We did not observe any degradation or change in sample color after heating to 1753 K. We used *E*_i_ = 40 and 30 meV at ARCS (phonon creation) and HYSPEC (phonon annihilation, see Supplementary Fig. 10 for full coverage) with energy resolution of ~1.8 and ~2.0 meV at the elastic line (FWHM), respectively. The triple-axis measurements at HB-3 were performed using the PG002 monochromator and analyzer, with a constant final energy *E*_f_ = 14.7 meV, and collimation settings of 48′–60′–60′–120′. Furthermore, our thermogravimetric measurements using TGA Q5000IR (0.1 mg sensitivity) in atmospheric conditions (106.054 mg sample in ceramic pan for 1140 min at 1473 K) did not show any observable mass loss, while similar measurements with Argon flow (154.139 mg sample in ceramic pan for 1140 min at 1473 K) had 0.6% mass loss. Although our single-crystal XRD measurements (not shown) with Argon flow showed similar results when compared to the results of atmospheric conditions.

### Harmonic density functional theory simulations

Phonon simulations were performed in the framework of DFT as implemented in the Vienna Ab initio Simulation Package (VASP 5.3)^44–46^. We used 4 × 4 × 2 gamma-centered Monkhorst–Pack electronic *k*-point mesh with a plane-wave cut-off energy of 500 eV in all of our simulations. The convergence criteria for electronic self-consistent loop was set to 10^−8^ eV. The projector-augmented wave potentials explicitly included 11 valence electrons for Y (4*s* ^2^4*p* ^6^5*s* ^2^4*d* ^1^), 13 for Mn (3*p* ^6^4*s* ^2^3*d* ^5^), and six for O (2*s* ^2^2*p* ^4^). All our calculations were spin polarized (collinear) with A-type antiferromagnetic order. We used the local spin-density approximation (LSDA) with A-type AFM order and a Hubbard correction^15^. To treat the localized *d*-electron states of Mn in LSDA + U calculations, the total energy expression was described as introduced by Dudarev et al.^47^ with on-site Coulomb interaction *U* = 8.0 eV and on-site exchange interaction *J* = 0.88 eV^15^. During the relaxation of the structure, the atomic positions were optimized until forces on all atoms were smaller than 1 meV Å^−1^. We used lattice parameters *a* = 6.148 Å and *c* = 11.493 Å. Phonon dispersions were calculated in the harmonic approximation, using the finite displacement approach as implemented in Phonopy^48^. The atomic forces were obtained with VASP from 20 independent atomic displacements. The phonon calculations used a 2 × 2 × 2 supercell of the hexagonal cell containing 240 atoms. The atomic displacement amplitude was 0.04 Å.

### Finite temperature AIMD simulations

AIMD simulations were performed at 1500 K on a 3 × 3 × 1 supercell of high-*T* hexagonal phase containing 90 atoms. We used experimental lattice parameters *a* = 3.619 Å, and *c* = 11.341 Å^31^. AIMD simulations were performed using NVT-ensemble with Nosé–Hoover thermostat (MDALGO = 2, SMASS = 0.92). We used a plane-wave cut-off energy of 800 eV with the Γ-point mesh for Brillouin zone integration. The simulations ran for about 3000 fs with a time step of 2 fs. The remaining AIMD simulation parameters were kept identical to 0 K DFT simulations. The trajectories were subsequently post-processed using TDEP code^41,49,50^ to obtain temperature-dependent effective potential surface and second-order force constants at 1500 K. Second-order force constants were used to obtain phonon dispersion along [*H*, 0, 10] direction (in the low-*T* FE unit cell notation) and phonon DOS as shown in Supplementary Fig. 5. The phonon energy of ~4.8 meV in AIMD simulations at 1500 K at K-point, i.e., (1, 0, 10) or (2, 0, 10) agree quite well with our experimental values of 3.41 ± 0.76 and 5.44 ± 0.22 meV at 1423 and 1753 K, respectively (Fig. 2). In addition, the shift in yttrium dominated ~15 meV and oxygen dominated ~78 meV phonon peak in 0 K DFT simulations to ~10 and ~70 meV, respectively, at 1500 K is consistent with experimental phonon DOS reported by Gupta et al.^35^

### Frozen phonon potential

Potential energy curves, as shown in Supplementary Fig. 6, were obtained by calculating energy for different amplitudes of K_3_ and ${\mathrm{\Gamma }}_2^ -$ lattice distortions from DFT simulations in 30 atom low-*T* notation unit cell. Since K_1_ and ${\mathrm{\Gamma }}_1^ +$ lattice distortions are stable in high-*T* phase and have comparatively smaller amplitude, we do not consider the coupling of K_1_ and ${\mathrm{\Gamma }}_1^ +$ with K_3_ lattice distortion. The energy of the crystal can now be written as a function of $Q_{{\mathrm{K}}_3}$ and $Q_{{\mathrm{\Gamma }}_2^ - }$ ^15^,

1 $$\begin{array}{*{20}{l}} {E\left( {Q_{{\mathrm{K}}_3},Q_{{\mathrm{\Gamma }}_2^ - }} \right)} \hfill & \hskip-8pt = \hfill &\hskip-7pt {\alpha Q_{{\mathrm{K}}_3}^2 + \beta Q_{{\mathrm{K}}_3}^4 + \gamma Q_{{\mathrm{\Gamma }}_2^ - }^2 + \delta Q_{{\mathrm{\Gamma }}_2^ - }^4} \hfill \\ {} \hfill & {} \hfill & { + \zeta Q_{{\mathrm{K}}_3}^2Q_{{\mathrm{\Gamma }}_2^ - }^2 + \eta Q_{{\mathrm{K}}_3}^3Q_{{\mathrm{\Gamma }}_2^ - }} \hfill \end{array}.$$

Potential energy curves were fitted using the above energy expression, and the corresponding fit is shown in Supplementary Fig. 6. Our parameters, *α* = −1.278 eV, *β* = 0.804 eV, *γ* = 0.015 eV, *δ* = 5.01 × 10^−4^ eV, *ζ* = 0.080 eV, and *η* = −0.227 eV are in excellent quantitative agreement with results of Fennie and Rabe^15^.

The *T*-dependent potential in the high-*T* PE phase for K_3_ lattice distortion is calculated from second-order force constants obtained from AIMD simulations at 1500 K. The potential curve at 1500 K can be expressed as $E\left( {Q_{{\mathrm{K}}_3},T} \right) = \alpha (T)Q_{{\mathrm{K}}_3}^2$ for *α*(*T* = 1500 K) = 1.47 eV. The potential for K_3_ distortion can be interpolated between the high-*T* PE and low-*T* FE phase to obtain the potential at the intermediate temperatures—one nearby *T*_FE_ where curvature in potential is close to zero and another for *T* < *T*_FE_. Linear interpolation of the potential between 0 and 1500 K leads to *T*_FE_ ≃ 710 K, while for quadratic interpolation (by constraining $\partial E{\mathrm{/}}\partial Q_{{\mathrm{K}}_3} = 0$ at 0 K) *T*_FE_ ≃ 1120 K, similar to the experimental value of ~1260 K. $Q_{{\mathrm{K}}_3}$ obtained from quadratic interpolation of the potential curves follows the similar trend as experimental data shown in Supplementary Fig. 8a except that *T*_FE_ values have small difference. Moreover, for both interpolation schemes, square root of the curvature—$\sqrt {\frac{{\partial ^2E}}{{\partial Q_{{\mathrm{K}}_3}^2}}} = \sqrt {K_{{\mathrm{K}}_3}^{{\mathrm{eff}}}} \propto \omega _{{\mathrm{K}}_3}^{{\mathrm{eff}}}$ at equilibrium position increases with decreasing *T* from *T*_FE_ to 0 K, thus leading to stiffening (increase) of phonon frequency as experimentally observed and shown in Supplementary Figs. 2h and 5a. Here $K_{{\mathrm{K}}_3}^{{\mathrm{eff}}}$ and $\omega _{{\mathrm{K}}_3}^{{\mathrm{eff}}}$ are effective stiffness and phonon energy of K_3_ lattice distortion.

Furthermore, for the ${\mathrm{\Gamma }}_2^ -$ distortion, $Q_{{\mathrm{\Gamma }}_2^ - }$ at equilibrium position can be calculated by solving $\partial E{\mathrm{/}}\partial Q_{{\mathrm{\Gamma }}_2^ - } = 0$ to obtain $Q_{{\mathrm{\Gamma }}_2^ - } \simeq \eta (T)Q_{{\mathrm{K}}_3}^3{\mathrm{/}}\left( {2\gamma + 2\zeta (T)Q_{{\mathrm{K}}_3}^2} \right)$. To simplify the expression of $Q_{{\mathrm{\Gamma }}_2^ - }$ in terms of $Q_{{\mathrm{K}}_3}$, the term involving *δ* have been left out owing to order of magnitude small value compared to other parameters. $Q_{{\mathrm{\Gamma }}_2^ - }(T)$ calculated from experimental values of $Q_{{\mathrm{K}}_3}$ is shown in Supplementary Fig. 8a. The *T*-dependence of *η*(*T*) and *ζ*(*T*) has been expressed using *c*_1_ exp[−*c*_2_(*T*/*T*_FE_)^2^], where *c*_1_ is *η* and *ζ* values at 0 K calculated using frozen phonon DFT simulations, and *c*_2_ is found to be 3.5. Here we note that function *c*_1_ exp[−*c*_2_(*T*/*T*_FE_)^2^] is chosen for its simplicity to develop qualitative understanding, and may be of different form in other geometric ferroelectrics. Additionally, from the temperature dependent $Q_{{\mathrm{K}}_3}$, $Q_{{\mathrm{\Gamma }}_2^ - }$, and *ζ*, square root of the curvature—$\sqrt {\frac{{\partial ^2E}}{{\partial Q_{{\mathrm{\Gamma }}_2^ - }^2}}} = \sqrt {K_{\Gamma _2^ - }^{{\mathrm{eff}}}} \propto \omega _{{\mathrm{\Gamma }}_2^ - }^{{\mathrm{eff}}}$ as a function of $Q_{{\mathrm{\Gamma }}_2^ - }$ and *T* is shown in Supplementary Fig. 8c, d, respectively. Here $K_{{\mathrm{\Gamma }}_2^ - }^{{\mathrm{eff}}}$ and $\omega _{{\mathrm{\Gamma }}_2^ - }^{{\mathrm{eff}}}$ are effective stiffness and phonon energy of ${\mathrm{\Gamma }}_2^ -$ distortion.

### Phonon intensity simulations

The simulated phonon intensity was calculated using the following expression:

2 $$\begin{array}{*{20}{l}} {S({\bf{Q}},E)} \hfill & \propto \hfill & {\mathop {\sum}\limits_s \mathop {\sum}\limits_\tau \frac{1}{{\omega _s}}\left| {\mathop {\sum}\limits_d \frac{{\overline {b_d} }}{{\sqrt {M_d} }}{\mathrm{exp}}\left( { - W_d} \right){\mathrm{exp}}(i{\bf{Q}} \cdot {\bf{d}})\left( {{\bf{Q}} \cdot {\bf{e}}_{{\mathrm{ds}}}} \right)} \right|^2} \hfill \\ {} \hfill & {} \hfill & { \times \left\langle {n_s + 1} \right\rangle \delta \left( {\omega - \omega _s} \right)\delta ({\bf{Q}} - {\bf{q}} - \tau )} \hfill \end{array}$$

3 $$\chi {\prime\prime}({\bf{Q}},E) = \frac{{S({\bf{Q}},E)}}{{n_s + \frac{1}{2} \pm \frac{1}{2}}},$$

where $\overline {b_d}$ is neutron scattering length, **Q** = **k** − **k**′ is the wave vector transfer, and **k**′ and **k** are the final and incident wave vector of the scattered particle, **q** the phonon wave vector, *ω*_*s*_ the eigenvalue of the phonon corresponding to the branch index *s*, ***τ*** is the reciprocal lattice vector, *d* the atom index in the unit cell, exp(−2*W*_*d*_) the corresponding DW factor, and $n_s = \left[ {{\mathrm{exp}}\left( {\frac{{\hbar \omega _s}}{{k_{\mathrm{B}}T}}} \right) - 1} \right]^{ - 1}$ is the mean Bose–Einstein occupation factor. The + and − sign in Eq. (3) correspond to phonon creation and phonon annihilation, respectively. The phonon eigenvalues and eigenvectors in Eq. (2) were obtained by solving dynamical matrix using Phonopy^48^.

### Data availability

The data that support the findings of this study are available from the corresponding author upon reasonable request.

## Electronic supplementary material

Supplementary Information
