# Supplementary material for: Momentum-resolved observations of the phonon instability driving geometric improper ferroelectricity in yttrium manganite
Source: Nat Commun. 2018 Jan 2;9:15. doi: 10.1038/s41467-017-02309-2 (PMC5750229; doi:10.1038/s41467-017-02309-2)
Supplement: Supplementary file 1 — Supplementary Information [file 41467_2017_2309_MOESM1_ESM.pdf]

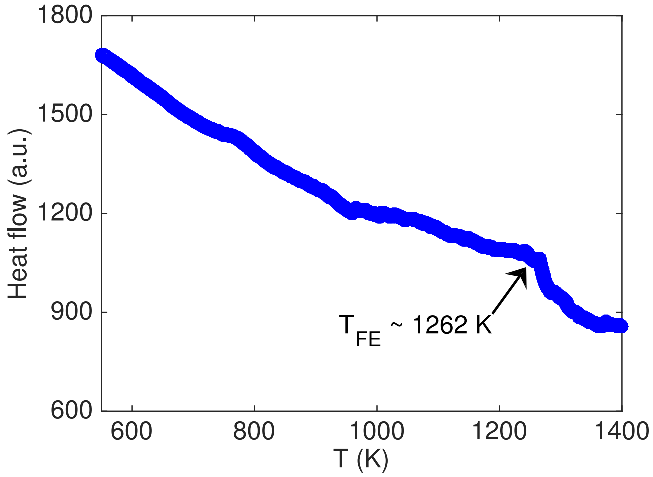

Supplementary Figure 1. Heat flow in $YMnO_3$ single-crystal of mass∼52.9 mg measured using NETZSCH DSC 404 F1 in Argon. The ferroelectric transition at 1262±5 K is marked by black arrow.

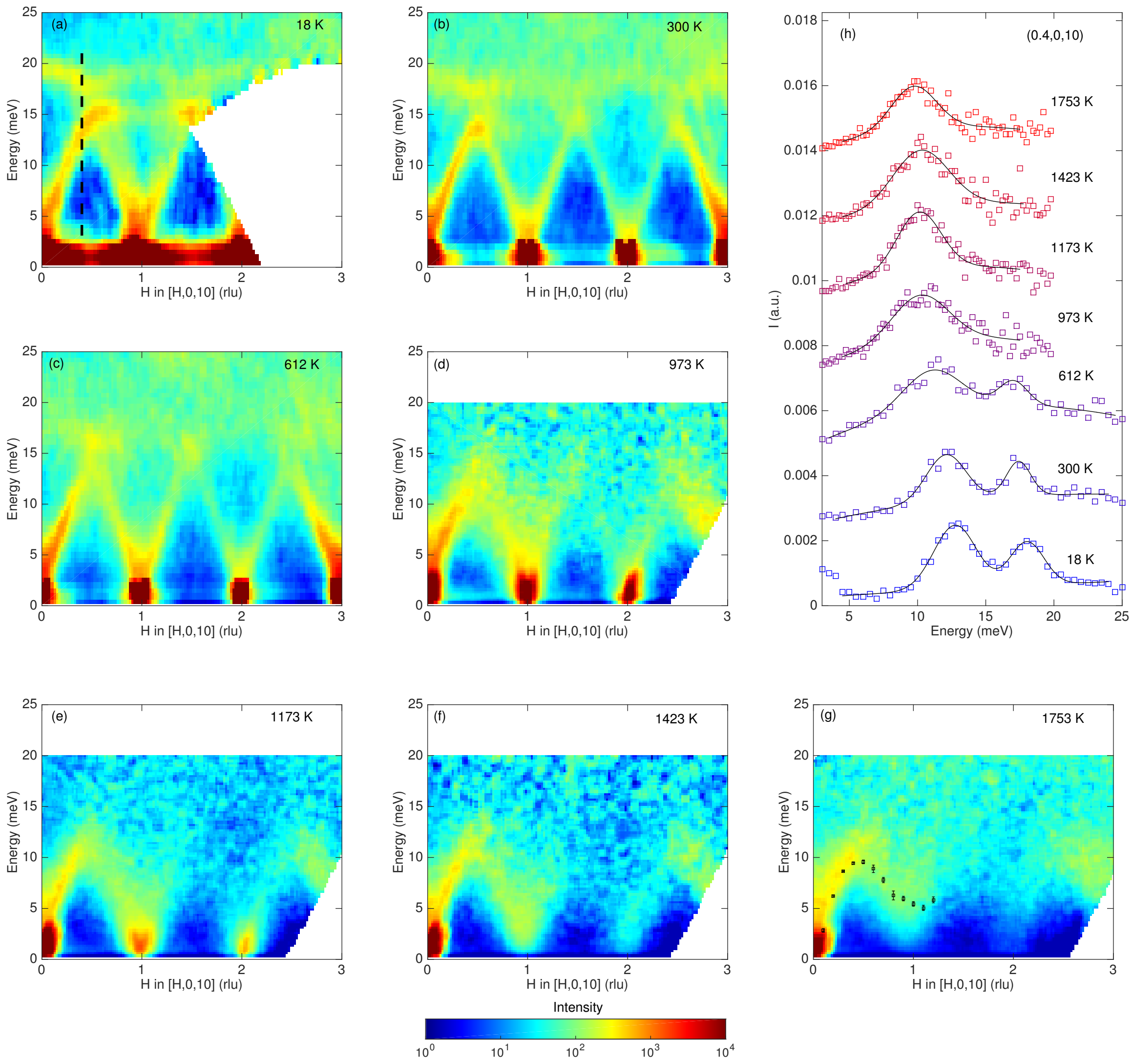

Supplementary Figure 2. Phonon dispersions showing the dynamics of K_3 distortion. (a-g) Phonon dispersion measured at T = 18, 300, 612, 973, 1173, 1423, and 1753 K. The Intensity is $\chi''(\mathbf{Q}, E) = S(\mathbf{Q}, E)/(n_s + \frac{1}{2} \pm \frac{1}{2})$ (see Methods section for details of the expression). In panel (g), the phonon dispersion obtained by fitting the intensity is shown as black markers with error-bars. (h) 1-D energy vs intensity cuts at HKL = (0.4,0,10) [as shown by black dotted line panel (a)] corresponding to TA phonon governing the K_3-like distortion. The intensity profile is fitted to Gaussian curves and over-plotted with experimental markers. The integration range in panel (a-g) along perpendicular Q directions is from -0.2 to 0.2 rlu, while for 1-D cuts, the integration range along H is from 0.35 to 0.45 rlu. To improve the statistics of image plots and 1-D cuts, data at equivalent q-point HKL = (H,0,10) is summed with data at HKL = (-H,0,10). Error bars in panel (h) are smaller than the size of markers. The data is offset for clarity. The data in panels (a-c) and (d-g) is measured on ARCS (phonon creation) and HYSPEC (phonon annihilation) spectrometer, respectively.

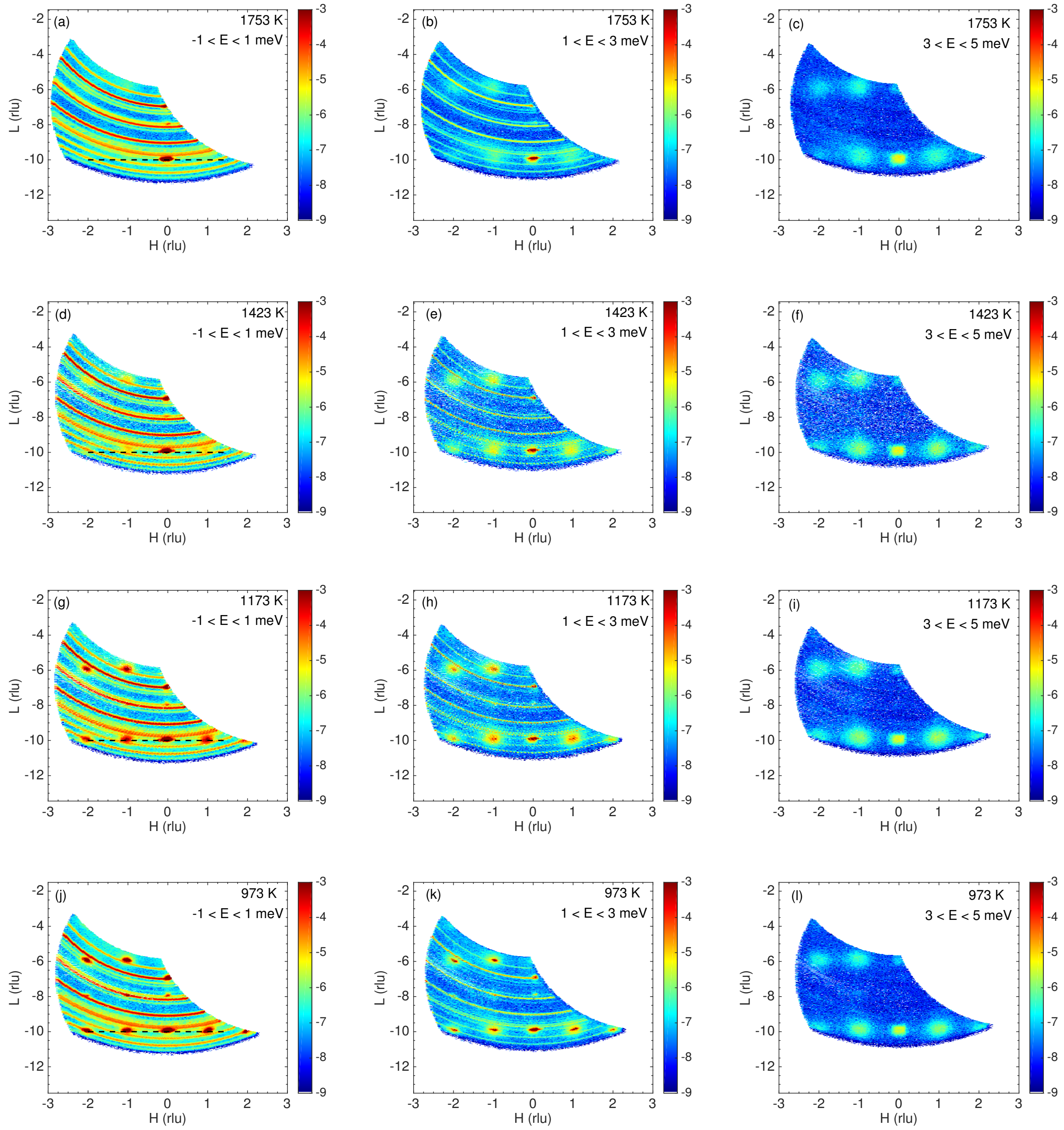

Supplementary Figure 3. Static and dynamical correlations near T_{FE}. Constant-E cuts of reciprocal space at (a-c) 1753 K, (d-f) 1423 K, (g-i) 1173 K, and (j-l) 973 K. At 1423 and 1753 K, elastic cuts [panel (a) and (d)] through reciprocal space show that Bragg peaks at (±1,0,10), (±2,0,10), (-1,0,6), and (-2,0,6) disappear; however dynamical correlations are still present, and these strong correlations persist up to at least 10 meV. Panels (b) and (e), and (c) and (f) show dynamical correlations between 1<E<3 and 3<E<5 meV above T_{FE}, respectively. Below T_{FE}, Bragg peaks (±1,0,10), (±2,0,10), (-1,0,6), and (-2,0,6) corresponding to condensation from K_3 and K_1 distortion appear, indicating the presence of strong, static correlations along with dynamical correlations. All cuts are integrated between -0.2 and 0.2 rlu in direction perpendicular to H and L. The Intensity shown is $S(\mathbf{Q})$.

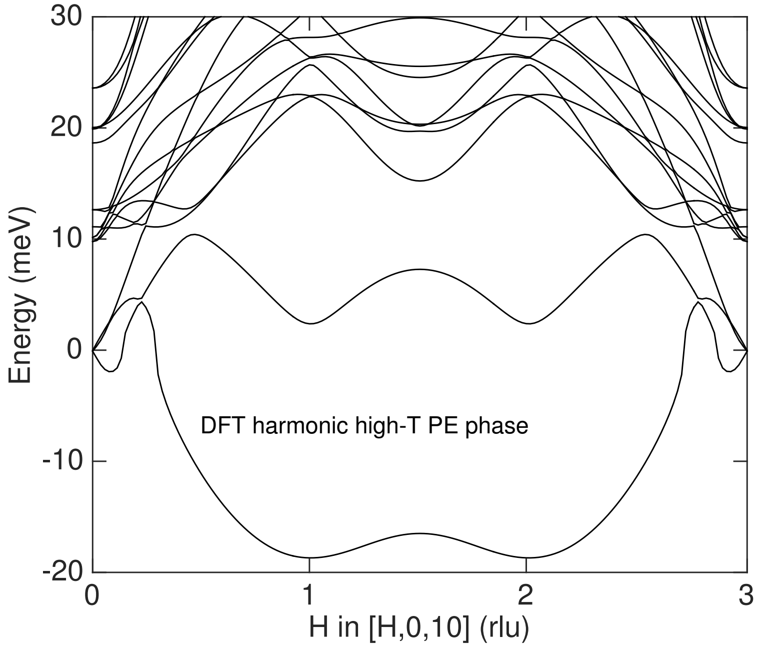

Supplementary Figure 4. Phonon dispersion along [H,0,10] and phonon DOS of $YMnO_3$ in high-T paraelectric phase calculated using DFT simulations at 0 K on $3\times3\times1$ supercell. The unstable phonons are anharmonically renormalized at finite temperature, and are shown in Supplementary Fig. 5(a).

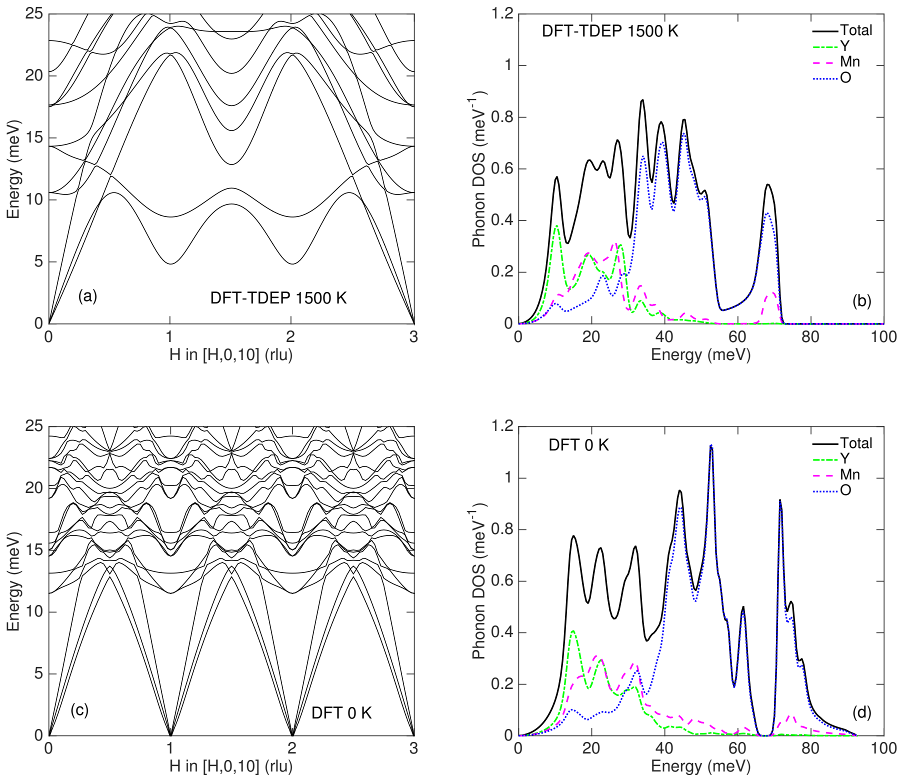

Supplementary Figure 5. Phonon simulations in the FE and PE phase. Phonon dispersion along [H,0,10] and phonon DOS of $YMnO_3$ in (a,b) high-T paraelectric and (c,d) low-T ferroelectric phase. AIMD simulations were performed for high-T phase at 1500 K, and subsequently post-processed using TDEP code to obtain phonon dispersion and DOS.

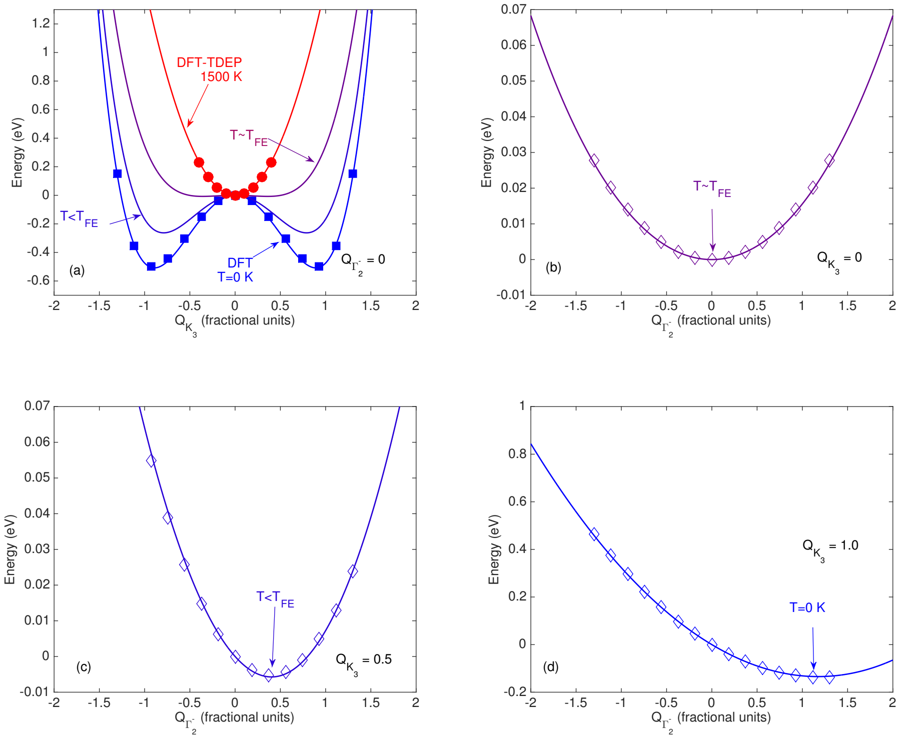

Supplementary Figure 6. Frozen phonon simulations of the energy surface. Energy as a function of (a) K_3 and (b) Γ_2^- lattice distortion. DFT-TDEP 1500 K is potential curve calculated from high-T PE phase force constants for K_3 lattice distortion, while intermediate curve at $T \sim T_{\mathrm{FE}}$ and $T < T_{\mathrm{FE}}$ are interpolation from 0 and 1500 K potential curves. (c,d) Energy as a function of Γ_2^- lattice distortion for a fixed amplitude of $Q_{\mathrm{K}_3} = 0.5$ and 1. The stable equilibrium position of Γ_2^- lattice distortion for different values of Q_{K_3} is marked with an arrow.

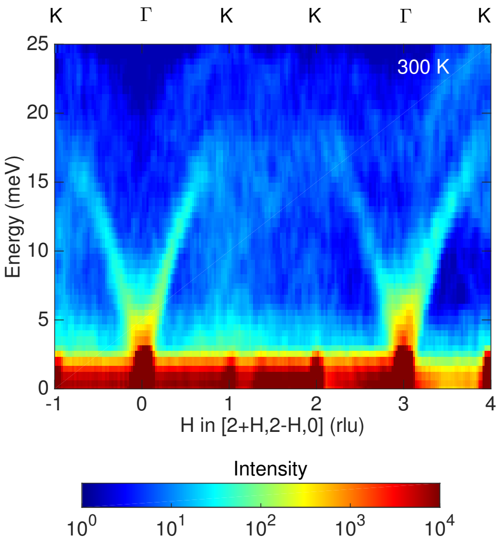

Supplementary Figure 7. Phonon dispersion of $YMnO_3$ along [2+H,2-H,0] direction at T = 300 K. Bragg peaks at (3,1,0), (1,3,0), (4,0,0), and (6,-2,0) are due to trimerization of unit cell. The integration range along perpendicular Q directions is from -0.2 to 0.2 rlu. The intensity I is $S(\mathbf{Q}, E)$. Γ and K symbols refer to high-T phase notation. The data is measured on ARCS instrument.

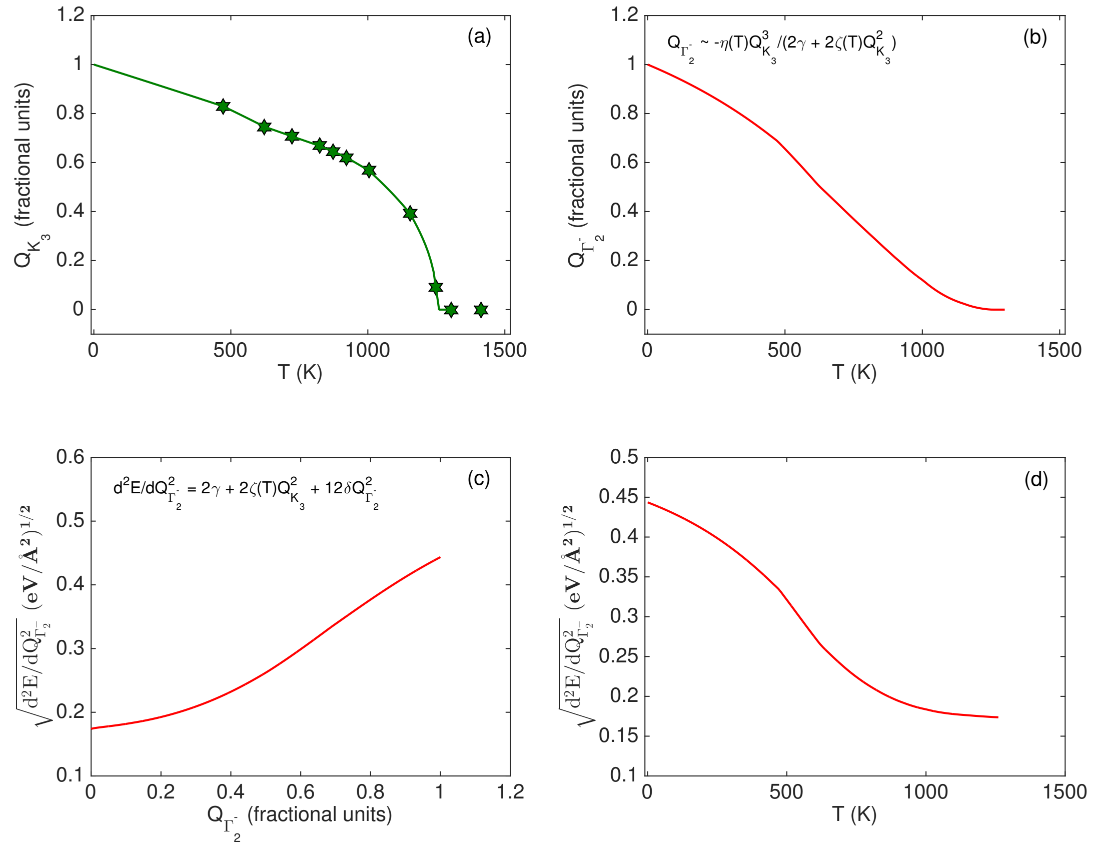

Supplementary Figure 8. Temperature dependence of K_3 and Γ_2^- distortions. (a) Q_{K_3} as a function of T obtained from single-crystal neutron diffraction measurements. The data is extrapolated to 0 K as shown by green solid line. (b) $Q_{\Gamma_2^-} \simeq \eta(T)Q_{K_3}^3/(2\gamma + 2\zeta(T)Q_{K_3}^2)$ as a function of T calculated using Q_{K_3} as shown in panel (a). (c) Square root of curvature – $\sqrt{\frac{\partial^2 E}{\partial Q_{\Gamma_2^-}^2}}$ as a function of $Q_{\Gamma_2^-}$. Since $\sqrt{\frac{\partial^2 E}{\partial Q_{\Gamma_2^-}^2}} = \sqrt{K_{\Gamma_2^-}^{eff}} \propto \omega_{\Gamma_2^-}^{eff}$, phonon frequency corresponding to Γ_2^- lattice distortion also increases with increasing amplitude of $Q_{\Gamma_2^-}$ [or equivalently decrease in T from $T_{\rm FE}$ to 0 K, panel (d)] as experimentally observed and shown in main text Fig. 5(a).

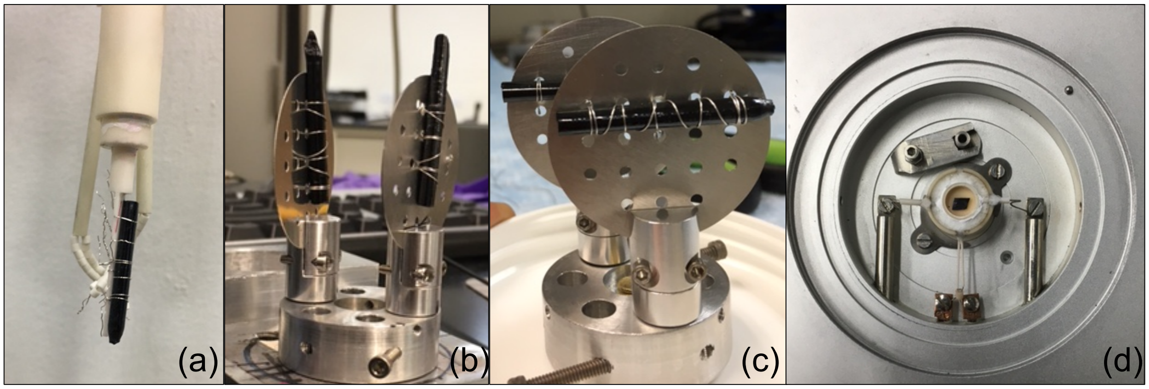

Supplementary Figure 9. Single-crystal samples for experimental measurements. (a) A single-crystal piece of mass∼3 gm mounted in H0L scattering plane on an Al_2O_3 post with Pt wires for HB-3 and HYSPEC instrument. Two single-crystal pieces, each of mass∼3 gm, co-aligned on an Al mount with Al wires in (b) H0L and (c) HK0 scattering plane for ARCS instrument. The mosaic of co-aligned samples was less than 1.5° in H0L and 2° in HK0 scattering plane. (d) A single-crystal piece of mass∼125 mg (dimension: $\sim 5 \times 5 \times 1$ mm) in a Linkam TS1500 stage at beamline 11-ID-C at the Advanced Photon Source.

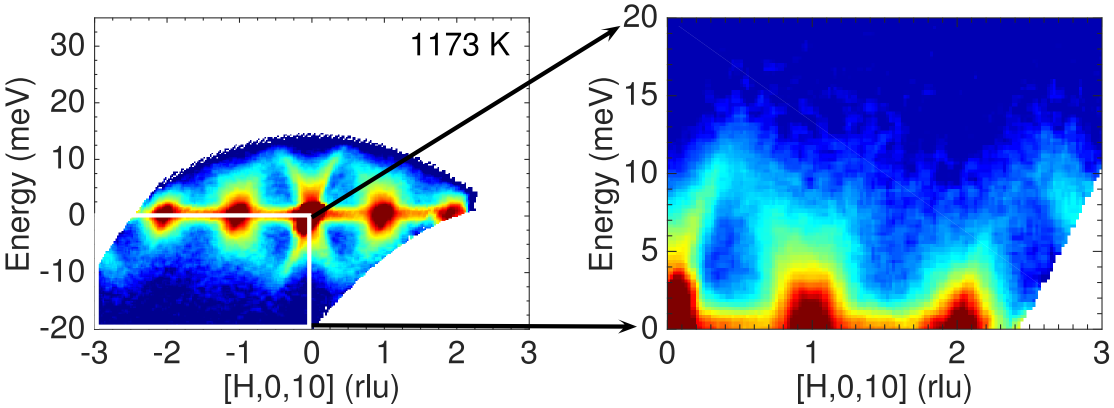

Supplementary Figure 10. Experimental $\mathbf{Q}-E$ coverage at HYSPEC instrument. Phonon dispersion of $YMnO_3$ along [H,0,10] direction, showing the $\mathbf{Q}-E$ coverage in phonon creation and phonon annihilation side (left panel), and phonon annihilation data shown in main text Fig. 2, and Supplementary Fig. 2 (right panel). The Intensity is $S(\mathbf{Q}, E)$.
